# Activation of the FAK-MAPK/ERK1 pathway promotes intestinal epithelial cell sheet migration during fistula tract repair

**DOI:** 10.1038/s41598-026-57499-x

**Published:** 2026-06-26

**Authors:** Cheng Wang, Cheng Geng, Shixing Wu, Jiangang Li, Junxiang Zhang, Baoqiang Xu, Bolin Zhang, Atigu Abuduwaili, Xinjian Xu

**Affiliations:** Department of Hepatobiliary and Pancreatic Surgery, The Fifth Affiliated Hospital of Xinjiang Medical University, Urumqi, 830000 Xinjiang Province China

**Keywords:** Focal adhesion kinase (FAK), MAPK/ERK1 signaling, Intestinal mucosa, Epithelial sheet migration, Fistula tract healing, Epithelial–mesenchymal transition (EMT), Cell biology, Diseases, Gastroenterology, Medical research

## Abstract

Postoperative fistula tracts remain a significant clinical challenge, yet the molecular mechanisms governing their healing are poorly understood. This study aimed to investigate the regulatory role of focal adhesion kinase (FAK) in intestinal mucosal epithelial cell migration during drainage tube-induced fistula tract healing and to elucidate its underlying mechanisms through the MAPK/ERK1 signaling pathway. A rabbit small intestinal fistula tract model was established and tissues were collected at 0, 7, 14, and 28 days post-surgery. Tissue characterization included histopathological evaluation (H&E and Masson trichrome staining) and molecular assessment of FAK phosphorylation, collagen deposition, and epithelial–mesenchymal transition (EMT) markers via immunohistochemistry, immunofluorescence, and Western blot analysis. In vitro, primary intestinal epithelial cells were isolated and FAK activity was modulated using pharmacological activation (ZINC40099027) or shRNA knockdown. Cell proliferation and migration were assessed via CCK-8, EdU, wound healing, and Transwell assays. Mechanistic involvement of the MAPK/ERK1 signaling pathway was validated using inhibitor (MAPK-IN-1) and agonist (C16-PAF) rescue experiments. In vivo, local injection of sh-FAK adenovirus evaluated FAK’s role in fistula tract healing. FAK phosphorylation (p-FAK) and Collagen I expression increased progressively during fistula tract maturation, exhibiting clear spatial co-localization in tissue sections. Pharmacological FAK activation enhanced epithelial cell proliferation, migration, MMP2/9 and Vimentin expression, and reduced E-cadherin levels, while shRNA-mediated FAK knockdown produced the opposite effects. Mechanistically, FAK activity positively correlated with MEK/ERK phosphorylation. Bidirectional rescue experiments using pathway inhibitors and agonists demonstrated that MAPK/ERK1 signaling is essential for FAK-induced cell migration. In vivo, local sh-FAK adenovirus injection suppressed MAPK/ERK1 activity, reduced granulation tissue and collagen deposition, and delayed fistula tract healing. FAK facilitates fistula tract repair by activating the MAPK/ERK1 pathway, driving epithelial cell proliferation, migration, and EMT-like transformation during the healing process. Targeting the FAK–MAPK/ERK1 axis represents a promising therapeutic strategy for managing refractory postoperative fistulas.

**Supplementary Information:**

The online version contains supplementary material available at https://doi.org/10.1038/s41598-026-57499-x.

## Introduction

In modern surgery, despite continuous advancements in surgical techniques, complications following complex abdominal procedures, such as pancreaticoduodenectomy, remain significant clinical challenges^1,2^. Among these, anastomotic leakage represents the primary contributor to high postoperative morbidity and mortality^3^. When anastomotic leakage develops, it is frequently complicated by intra-abdominal infections and abscess formation, which are conventionally managed via drainage tube placement^4^. However, prolonged indwelling drainage can lead to the formation of a fibrotic channel connecting the internal cavity to the external surface, known as a “fistula tract”^5^. The formation and eventual resolution of fistula tracts involve dynamic and complex biological processes analogous to classical tissue repair, characterized by precisely orchestrated stages of inflammation, cell proliferation and migration, extracellular matrix (ECM) deposition, and tissue remodeling^6^. A pivotal event in tract closure is the coordinated migration of intestinal mucosal epithelial cells as cohesive sheets along the surface of newly deposited granulation tissue—referred to as “epithelial sheet migration”—which re-establishes the epithelial barrier lining the fistula tract^7–9^. This re-epithelialization is the decisive factor determining successful fistula closure and restoration of functional integrity. Understanding the molecular mechanisms that regulate epithelial sheet migration therefore holds considerable scientific importance for tissue repair biology and offers a rational basis for designing novel therapeutic approaches to enhance healing of clinically refractory fistulas.

Focal Adhesion Kinase (FAK) is a non-receptor tyrosine kinase localized at focal adhesions, serving as a central hub for integrating intracellular and extracellular signals^10^. FAK can be activated by integrin-mediated ECM engagement as well as by growth factors (e.g., EGF, PDGF), thereby transducing extracellular physical and chemical cues into intracellular responses, regulating cytoskeletal dynamics, and ultimately influencing fundamental cellular processes including proliferation, survival, differentiation, and migration^11–14^. Extensive evidence demonstrates that FAK plays pivotal roles in various physiological and pathological processes. In skin wound healing, FAK activation promotes keratinocyte migration^15^; in fibrotic diseases such as renal fibrosis and hypertrophic scarring, aberrant FAK activation drives excessive fibroblast proliferation and ECM deposition^16,17^. In the context of cancer, FAK is recognized as a critical driver of tumor invasion and metastasis, with its expression levels correlating closely with tumor malignancy and patient prognosis^18,19^. Given that epithelial cell sheet migration represents a highly coordinated collective migratory process, and FAK serves as a key regulatory node in cellular migration networks^9^, it is reasonable to hypothesize that FAK may similarly influence intestinal mucosal injury repair, especially within the unique microenvironment of drainage tube-induced fistula tracts. However, despite its established roles in other tissues, the specific functions and regulatory mechanisms of FAK in intestinal mucosal repair and fistula tract healing remain largely unexplored.

The Mitogen-Activated Protein Kinase (MAPK) signaling pathway is a fundamental signal transduction cascade in eukaryotic cells that transmits extracellular stimuli to the nucleus to regulate gene expression and cellular responses^20^. Among the MAPK family members, the classical MAPK/ERK pathway (ERK1/2) is the most extensively studied and critically regulates cell proliferation, differentiation, migration, and survival. Importantly, FAK can function as an upstream activator of this pathway by recruiting and activating Ras through adaptor proteins (e.g., Grb2/Sos), thereby initiating the sequential Raf-MEK-ERK phosphorylation cascade that culminates in ERK1/2 activation^21^. This “FAK-MAPK/ERK1” signaling axis has been demonstrated to mediate migration in various cell types across different tissue contexts. However, whether FAK utilizes this specific pathway to promote intestinal epithelial sheet migration and thereby facilitate fistula tract healing remains a critical unresolved question that warrants investigation.

Based on this background, we propose the central hypothesis that during healing of drainage tube-induced fistula tracts, activated FAK triggers downstream MAPK/ERK1 signaling through phosphorylation, thereby promoting proliferation and epithelial sheet migration of intestinal mucosal epithelial cells and ultimately accelerating re-epithelialization of the fistula tract. To systematically test this hypothesis, we designed an integrated experimental framework combining in vivo and in vitro approaches: First, using a rabbit small intestinal fistula tract model, we examined FAK expression and activation patterns and their temporal correlation with tract healing dynamics at the tissue level. Second, through in vitro cell-based experiments involving pharmacological modulation and genetic manipulation, we dissected the direct effects of FAK on intestinal epithelial cell proliferation and migration. Finally, bidirectional rescue experiments using selective pathway inhibitors and agonists were employed to establish the essential mediating role of MAPK/ERK1 signaling in FAK-driven epithelial migration. This comprehensive study aims to provide novel molecular targets and mechanistic insights for developing therapeutic strategies to enhance postoperative fistula tract repair.

## Materials and methods

### Experimental animals and model establishment

Animal care and housing

Thirty-two healthy adult male Chinese white rabbits (weighing 2.0–2.5 kg and aged 12–16 weeks) were purchased from Shanghai Slac Laboratory Animal Co., Ltd. (Shanghai, China). All animals were housed individually in stainless steel cages and maintained in a specific pathogen-free (SPF) environment under controlled conditions: temperature 22 ± 2 °C, relative humidity 50 ± 10%, 12-h light/dark cycle, and adequate ventilation. Rabbits had ad libitum access to standard laboratory chow and sterile water. After one week of acclimation, all experimental procedures were performed in strict accordance with the Guidelines for the Care and Use of Laboratory Animals and were approved by the Experimental Animal Ethics Committee of The Fifth Affiliated Hospital of Xinjiang Medical University.

#### Surgical procedure for fistula tract model establishment

Prior to surgery, rabbits were fasted for 12 h with free access to water. General anesthesia was induced via intravenous injection of 3% sodium pentobarbital (1 mL/kg) into the marginal ear vein. Anesthetic depth was monitored by assessing pedal withdrawal reflex, respiratory rate, and heart rate. The animals were secured in a supine position, and body temperature was maintained at 37–39 °C using a heating pad. The abdominal area was shaved, prepared with 75% ethanol and 10% povidone-iodine solution, and draped under aseptic conditions. A midline laparotomy incision (approximately 5 cm) was made to enter the abdominal cavity through the linea alba. The ileum was identified approximately 15 cm proximal to the ileocecal junction. After temporarily occluding the blood supply with atraumatic intestinal clamps, the selected segment was transected at its midpoint, and an end-to-side anastomosis was performed using continuous 5 − 0 absorbable sutures (polyglactin 910) with an inverting seromuscular technique.

A small incision (approximately 0.5 cm) was then created on the antimesenteric border of the distal loop, and an 8 F silicone drainage tube was inserted approximately 5 cm into the lumen and secured with a purse-string suture to prevent displacement. The tube was externalized through a separate stab incision in the right lower abdominal wall, and its distal end was securely ligated and closed without connection to a drainage bag to simulate a closed fistula tract. The abdominal wall was closed in layers using 3 − 0 absorbable sutures for the peritoneum and muscle layers, and 4 − 0 non-absorbable sutures for the skin. Sham-operated rabbits underwent laparotomy and gentle intestinal manipulation without anastomosis or drainage tube insertion, followed by closure in the same manner.

#### Postoperative care and sample collection

Postoperatively, rabbits were placed in individual recovery cages with supplemental heating until full recovery from anesthesia. Benzylpenicillin (400,000 U/rabbit) was administered intramuscularly once daily for three consecutive days to prevent infection. Rabbits were monitored daily for signs of distress, weight loss, wound complications, or infection. Analgesics (buprenorphine, 0.05 mg/kg, subcutaneously, twice daily for 3 days) were administered to minimize pain. Rabbits were euthanized at 6 h (designated day 0), 7, 14, and 28 days post-surgery (*n* = 8 per time point) by intravenous overdose of 3% sodium pentobarbital (150 mg/kg, marginal ear vein) until respiratory arrest and loss of pedal withdrawal and corneal reflex were confirmed. Death was verified by the absence of heartbeat and respiration for a minimum of 5 min in accordance with the AVMA Guidelines for the Euthanasia of Animals (2020). Fistula tract tissues surrounding the drainage tube were aseptically dissected under sterile conditions. Portions of each tissue sample were snap-frozen in liquid nitrogen and stored at − 80 °C for subsequent protein and RNA extraction, while the remaining specimens were immersed in 4% paraformaldehyde in phosphate-buffered saline (PBS, pH 7.4) for at least 24 h at 4 °C for histopathological and immunological analyses. Fixed tissues were then dehydrated through graded ethanol series, cleared in xylene, and embedded in paraffin for sectioning.

### Histological and immunostaining analysis

Fixed fistula tract tissues were dehydrated through a graded ethanol series, cleared in xylene, embedded in paraffin, and sectioned at 4 μm thickness. Deparaffinized and rehydrated sections were stained with Hematoxylin and Eosin (H&E) or Masson’s Trichrome to evaluate tissue architecture, cellular composition, and collagen deposition. For immunohistochemistry, antigen retrieval was performed by heating sections in citrate buffer (pH 6.0, 10 mM) at 95 °C for 20 min, followed by quenching of endogenous peroxidase activity with 3% hydrogen peroxide for 15 min and blocking with 5% bovine serum albumin in PBS for 30 min at room temperature. Sections were then incubated overnight at 4 °C in a humidified chamber with primary antibodies including rabbit anti-phospho-FAK (Tyr397) (ABclonal, Cat. No. AP1547, 1:200), rabbit anti-Collagen I (ABclonal, Cat. No. A24863, 1:500), rabbit anti-myeloperoxidase (MPO) (Abcam, Cat. No. ab208670, 1:200), or rabbit anti-CD90/Thy1 (Abcam, Cat. No. ab307736, 1:150). Following three washes in PBS, HRP-conjugated goat anti-rabbit secondary antibodies (Servicebio, Cat. No. GB23303, 1:200) were applied for 1 h at room temperature, and immunoreactivity was visualized using DAB substrate with hematoxylin counterstaining. For immunofluorescence, sections underwent the same antigen retrieval and blocking steps, followed by permeabilization with 0.3% Triton X-100 for 15 min and overnight incubation at 4 °C with combinations of rabbit anti-phospho-FAK (1:200) and mouse anti-Collagen I (ABclonal, Cat. No. A24863, 1:500). After washing, sections were incubated with Alexa Fluor 594-conjugated goat anti-rabbit IgG (Invitrogen, Cat. No. A11012, 1:500) and Alexa Fluor 488-conjugated goat anti-mouse IgG (Invitrogen, Cat. No. A11001, 1:500) for 1 h at room temperature in darkness, mounted with DAPI-containing antifade medium, and imaged using a confocal laser scanning microscope (Zeiss LSM 880). At least five randomly selected fields per section from three independent samples were acquired and quantitatively analyzed using ImageJ and ZEN software.

### Primary intestinal epithelial cell isolation, culture, and experimental treatments

Intestinal mucosal tissues were aseptically collected from healthy newborn rabbits (aged 1–3 days), rinsed three times with ice-cold PBS containing antibiotics (100 U/mL penicillin and 100 µg/mL streptomycin), and the mucosal layer was gently scraped using a sterile glass slide, minced into 1–2 mm³ fragments, and digested with 0.25% trypsin-EDTA (Gibco, Cat. No. 25200056) for 30 min at 37 °C with continuous gentle shaking (100 rpm). Digestion was terminated by adding an equal volume of high-glucose DMEM (Gibco, Cat. No. 11965092) supplemented with 10% fetal bovine serum (FBS, Gibco, Cat. No. 10099141). The cell suspension was filtered through a 100 μm nylon cell strainer (Falcon, Cat. No. 352360) and centrifuged at 300 × g for 5 min at 4 °C to collect the pellet, which was resuspended in complete DMEM containing 10% FBS and 1% penicillin-streptomycin (Gibco, Cat. No. 15140122), and seeded into tissue culture-treated dishes at a density of 1 × 10⁶ cells/mL. Cells were cultured at 37 °C in a humidified atmosphere with 5% CO₂, during which epithelial cells preferentially adhere to tissue culture-treated surfaces due to their integrin-mediated adhesion properties, while contaminating fibroblasts and other stromal cells exhibit slower attachment kinetics. After this 2-hour incubation period, non-adherent and loosely adherent cells (primarily fibroblasts and immune cells) were removed by gentle washing with PBS, effectively enriching the epithelial population. Culture medium was changed every 2–3 days, and cells at passages 2–3 were used for all experiments. Epithelial identity was confirmed by immunofluorescence staining for cytokeratin 18 (CK18, a specific marker of intestinal epithelial cells; ABclonal, Cat. No. A20104, 1:200) following fixation with 4% paraformaldehyde for 15 min and permeabilization with 0.1% Triton X-100 in PBS for 10 min, followed by blocking with 5% BSA for 1 h and incubation with primary antibody overnight at 4 °C. After washing, cells were incubated with Alexa Fluor 488-conjugated secondary antibody (1:500) for 1 h, counterstained with DAPI, and imaged by fluorescence microscopy. Cells showing > 95% CK18 positivity were considered pure epithelial cultures. Experimental treatments included a control group (untreated cells cultured in complete medium), a FAK agonist group treated with ZINC40099027 (MedChemExpress, Cat. No. HY-134570, 500 nM for 24 h), lentiviral transduction groups with non-targeting control shRNA (sh-NC) or FAK-targeting shRNA (sh-FAK; GeneChem, Shanghai, China) at a multiplicity of infection (MOI) of 20 with 5 µg/mL polybrene for 24 h, followed by selection with 2 µg/mL puromycin for 72 h to establish stable knockdown cell lines, and rescue experiments in which a MAPK pathway inhibitor (U0126, MEK1/2 inhibitor, MedChemExpress, Cat. No. HY-12031 A, 1 µM) or agonists (C16-PAF, a potent MAPK and MEK/ERK activator, MedChemExpress, Cat. No. HY-108635, 5 µM) were applied for 24 h in combination with FAK modulation to elucidate pathway-specific effects on cell proliferation and migration.

### Cell viability and proliferation assays

Cell viability was assessed using the Cell Counting Kit-8 (CCK-8) assay (Dojindo, Cat. No. CK04). Cells after each treatment were seeded in 96-well plates at a density of 5 × 10³ cells per well in 100 µL complete medium and allowed to attach overnight. Then 10 µL of CCK-8 solution was added to each well, and plates were incubated for 2 h at 37 °C in the dark. Absorbance at 450 nm was measured using a microplate reader (BioTek Synergy H1, USA), with each treatment performed in six replicate wells. Cell viability was calculated as a percentage relative to the control group. Cell proliferation was evaluated using the 5-ethynyl-2’-deoxyuridine (EdU) incorporation assay (RiboBio, Cat. No. C10310). Cells were seeded in 96-well plates at 5 × 10³ cells per well, treated for 24 h, then incubated with 10 µM EdU for 2 h at 37 °C. After fixation with 4% paraformaldehyde for 15 min and permeabilization with 0.3% Triton X-100 for 10 min, EdU-positive cells were detected using Click-iT reaction cocktail according to the manufacturer’s protocol. Nuclei were counterstained with DAPI, and five random fields per well were captured using a fluorescence microscope (Nikon Eclipse Ti-U). The EdU incorporation rate was calculated as the percentage of EdU-positive cells relative to total DAPI-stained cells using ImageJ software.

### Wound healing and transwell migration assays

Wound healing assays were performed by seeding cells in 6-well plates at 5 × 10⁵ cells per well and growing them to confluence over 24 h. Following treatment application, a linear scratch wound was created across the cell monolayer with a sterile 200 µL pipette tip. Dislodged cells were immediately washed away with PBS, and the medium was replaced with serum-free medium to minimize proliferation-mediated gap closure. Images of the same marked positions were captured at 0 and 24 h under an inverted microscope (Nikon Eclipse TS100) at 100× magnification, and the wound closure rate was quantified using ImageJ software by measuring the remaining gap area and calculating: Wound closure (%) = [(Area₀ₕ − Area₂₄ₕ) / Area₀ₕ] × 100%. For Transwell migration assays, logarithmic-phase cells were trypsinized, counted, and resuspended in serum-free medium, then seeded into the upper chamber of Transwell inserts (8 μm pore size, Corning, Cat. No. 3422) at 5 × 10⁴ cells in 200 µL, with 600 µL of medium containing 10% FBS added to the lower chamber as a chemoattractant. After incubation for 24 h at 37 °C in a 5% CO₂ incubator, non-migrated cells on the upper membrane surface were gently removed using a cotton swab, and migrated cells on the lower membrane surface were fixed with ice-cold methanol for 15 min and stained with 0.1% crystal violet for 20 min at room temperature. After washing with PBS and air-drying, inserts were photographed, and five random fields per insert were imaged at 200× magnification, and migrated cells were counted using ImageJ software. Each experiment was performed in triplicate wells and repeated three times independently.

### Western blot analysis

Total protein was extracted from tissues or cultured cells using RIPA lysis buffer (Beyotime, Cat. No. P0013B) containing protease and phosphatase inhibitors (Roche, Cat. No. 04693159001 and 04906837001, respectively). Lysates were incubated on ice for 30 min with periodic vortexing, then centrifuged at 12,000 × g for 15 min at 4 °C to remove cellular debris. Protein concentrations in the supernatants were determined by the BCA assay (Thermo Fisher, Cat. No. 23227). Equal amounts of protein (30 µg) were mixed with 5× SDS loading buffer, denatured at 95 °C for 5 min, and separated by 10% or 12% SDS-PAGE at 120 V for approximately 90 min and transferred onto PVDF membranes (Millipore, Cat. No. IPVH00010) at 300 mA for 90 min at 4 °C using a wet transfer system. Membranes were blocked with 5% non-fat milk or 5% BSA in TBST (Tris-buffered saline with 0.1% Tween-20) for one hour at room temperature and incubated overnight at 4 °C with gentle shaking with primary antibodies, including FAK (ABclonal, A11195, 1:1000), p-FAK (ABclonal, AP1547, 1:1000), E-cadherin (ABclonal, A11492, 1:1000), Vimentin (ABclonal, A2584, 1:1000), MMP2 (ABclonal, A6247, 1:1000), MMP9 (ABclonal, A26079, 1:1000), MEK1/2 (ABclonal, A24394, 1:1000), p-MEK1/2 (ABclonal, AP0063, 1:1000), Erk1/2 (ABclonal, A4782, 1:1000), p-Erk1/2 (ABclonal, AP0472, 1:1000), and β-actin (ABclonal, #4967, 1:20,000). Membranes were washed three times with TBST and incubated with HRP-conjugated goat anti-rabbit or anti-mouse secondary antibodies (Servicebio, Cat. No. GB23303 or GB23301, 1:5000) for one hour at room temperature. Protein bands were visualized using ECL chemiluminescent substrate (Thermo Fisher, Cat. No. 32106) and imaged using a chemiluminescence imaging system (Bio-Rad ChemiDoc XRS+, USA). Band intensities were quantified with ImageJ software, and protein expression levels were normalized to β-actin. Phosphorylated protein levels were expressed as the ratio of phosphorylated to total protein.

### In vivo adenoviral gene transfer

Concurrent with the establishment of the rabbit fistula model, local gene silencing was performed via direct tissue injection of adenoviral vectors. Following drainage tube placement and securing, a sterile 1 mL microsyringe with a 30-gauge needle was used to perform multiple uniform injections (approximately 8–10 injection sites, 50 µL per site, total volume 500 µL) of either sh-NC (non-targeting control shRNA) or sh-FAK adenovirus (targeting rabbit FAK gene, GeneChem, Shanghai, China; 1 × 10⁹ plaque-forming units [PFU] per rabbit) into the intestinal wall and adjacent mesenteric tissues within a 1 cm radius surrounding the drainage tube to ensure comprehensive transduction of cells in the fistula tract microenvironment. Injections were performed carefully to avoid intraluminal leakage or vascular injury. Animals were housed postoperatively under standard conditions as described above (Sect.  2.1) for 2 weeks, during which they received routine monitoring and antibiotic prophylaxis, after which they were euthanized by intravenous overdose of 3% sodium pentobarbital (150 mg/kg) as previously described, and fistula tract tissues were aseptically collected for histological and immunological analyses. A minimum of six animals per group were used for statistical analysis.

### Enzyme-linked immunosorbent assay (ELISA)

Inflammatory cytokine levels in serum were measured using commercially available ELISA kits according to the manufacturers’ protocols. Blood samples (approximately 2–3 mL) were collected from the marginal ear vein at designated time points (days 0, 7, 14, and 28 post-surgery) and allowed to clot at room temperature for 30 min. Samples were then centrifuged at 3,000 × g for 15 min at 4 °C to obtain serum, which was aliquoted and stored at − 80 °C until analysis. Inflammatory cytokine levels were measured using commercially available ELISA kits according to the manufacturers’ protocols. Kits for TNF-α (A0277), IL-1β (A16288), IL-6 (A22222), IL-4 (A14660), and IL-10 (A2171) were purchased from ABclonal. Serum samples and standards were added to pre-coated 96-well plates in duplicate and processed following the manufacturer’s instructions. Absorbance was measured at 450 nm using a microplate reader (BioTek Synergy H1, USA), and cytokine concentrations were calculated from standard curves and expressed as picograms per milliliter (pg/mL).

### Statistical analysis

All experimental data were analyzed and graphed using GraphPad Prism 8.0 software. Normality of data distribution was assessed using the Shapiro–Wilk test, and all experimental data were confirmed to follow a normal distribution prior to statistical analysis. Continuous data are presented as mean ± standard deviation (mean ± SD). Comparisons between two groups were performed using unpaired Student’s t-test, while comparisons among multiple groups were conducted using one-way analysis of variance (ANOVA) followed by Tukey’s post hoc test. A P value < 0.05 was considered statistically significant. All in vitro experiments were performed in at least three independent biological replicates, and in vivo experiments included a minimum of six animals per group unless otherwise specified.

## Results

### Dynamic evolution of rabbit drainage tube-induced fistula tract and FAK activation

**Fig. 1:**
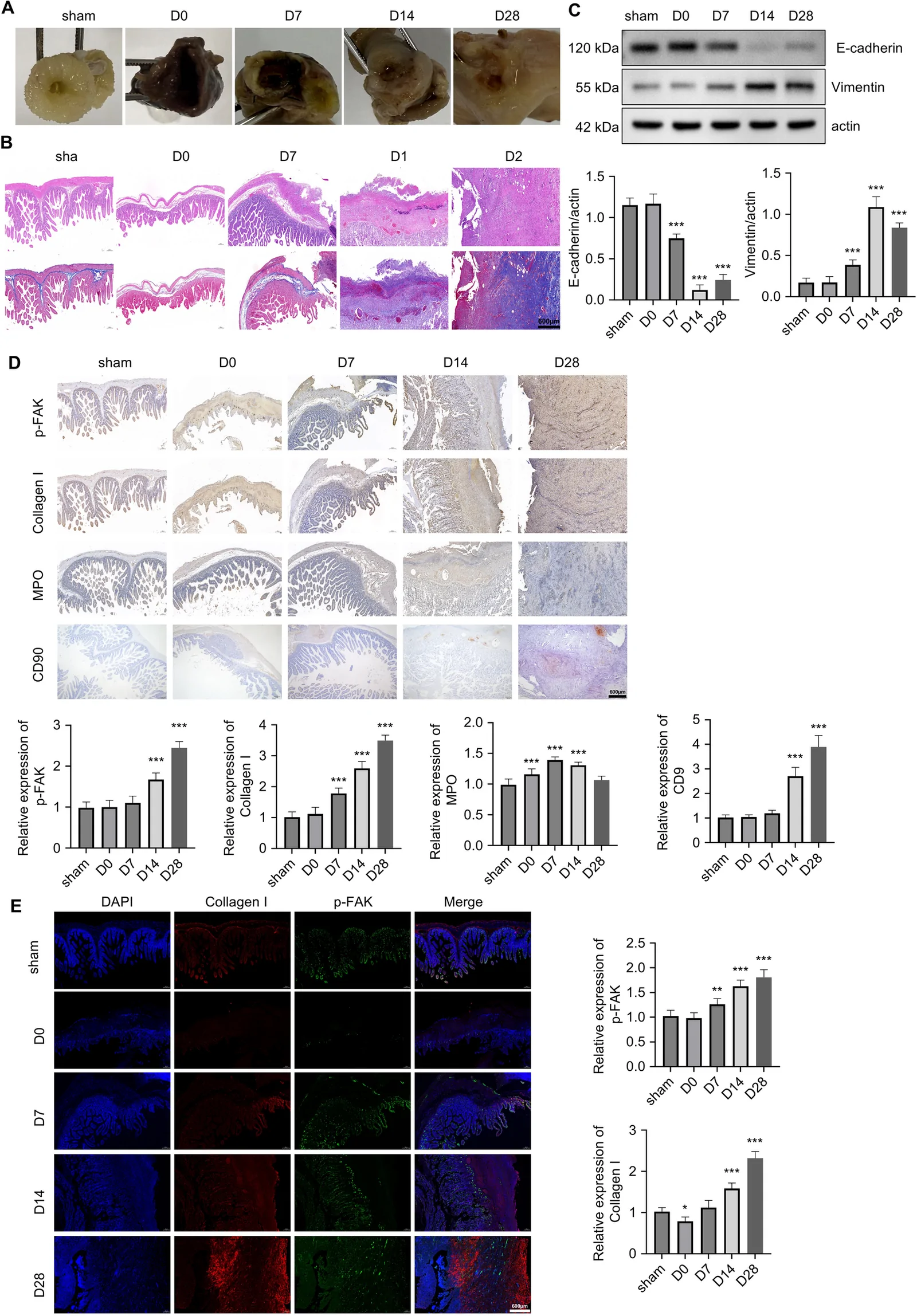
Dynamic evolution of rabbit drainage tube-induced fistula tract and FAK activation. (**A**) Gross morphology of fistula tracts at days 0, 7, 14, and 28 post-surgery, showing progressive fibrous encapsulation around the drainage tube. (**B**) Representative images of H&E staining, immunohistochemical staining for MPO and CD90, and Masson’s trichrome staining of fistula tract tissues at different time points. Scale bars = 50 μm. (**C**) Western blot analysis of E-cadherin and Vimentin expression in fistula tract tissues at days 0, 7, 14, and 28, with quantification shown in bar graphs. (**D**) Western blot analysis of p-FAK, total FAK, and Collagen I expression in fistula tract tissues at different time points, with quantification of p-FAK/FAK ratio and Collagen I levels. (**E**) Representative immunofluorescence images showing co-localization of p-FAK (red) and Collagen I (green) in fistula tract tissues at day 28. Nuclei were counterstained with DAPI (blue). Merged images show overlapping signals (yellow) in fibrotic regions. Scale bars = 600 μm. Data are presented as mean ± SD (*n* = 6 per group). **P < 0.01, ***P < 0.001 vs. day 0.

A reproducible rabbit small intestinal fistula tract model was successfully established permanent drainage tube placement^22^. Gross examination revealed progressive fibrous encapsulation around the drainage tube over time, evolving from initial inflammatory exudation at early time points to a mature, well-defined fibrous canal by 28 days (Fig. 1A). Histological analyses further demonstrated this dynamic temporal process. H&E staining indicated that early-stage tissues (days 0 and 7) exhibited massive infiltration of inflammatory cells with a disorganized tissue architecture, whereas later stages (days 14 and 28) showed a marked reduction in inflammatory cells, accompanied by proliferating fibroblasts and neovascularization, forming dense granulation tissue that progressively matured into organized fibrous tissue (Fig. 1B). Immunohistochemical staining for myeloperoxidase (MPO), a neutrophil marker, confirmed the temporal pattern of inflammation, showing strong MPO-positive cell infiltration at days 0 and 7, which decreased substantially by days 14 and 28. Conversely, CD90 (Thy-1), a fibroblast marker, exhibited minimal expression at early time points but increased progressively at days 14 and 28, indicating robust fibroblast recruitment and activation during fistula tract maturation (Fig. 1B). Masson’s trichrome staining revealed a progressive increase in collagen fiber deposition, with fibers transitioning from sparse and randomly oriented at early stages to increasingly organized and densely packed at later stages, forming the structural scaffold of the fistula tract wall (Fig. 1B). Western blot analysis demonstrated a time-dependent decrease in the epithelial marker E-cadherin and a concomitant increase in the mesenchymal marker Vimentin, suggesting an epithelial-mesenchymal transition (EMT)-like process occurring in epithelial cells within the fistula tract wall (Fig. 1C). Additionally, protein levels of activated FAK (p-FAK) and Collagen I increased significantly over time, peaking at days 14 and 28 (Fig. 1D). Immunofluorescence co-localization analysis confirmed the spatial association of p-FAK (red) and Collagen I (green) in mature fistula tract tissues at day 28, with merged images revealing substantial overlap predominantly in the fibrotic regions, indicating that FAK activation is closely linked to extracellular matrix remodeling and fibrosis during fistula tract formation (Fig. 1E).

### p-FAK is highly expressed during the proliferation-remodeling phase of fistula tract healing

**Fig. 2:**
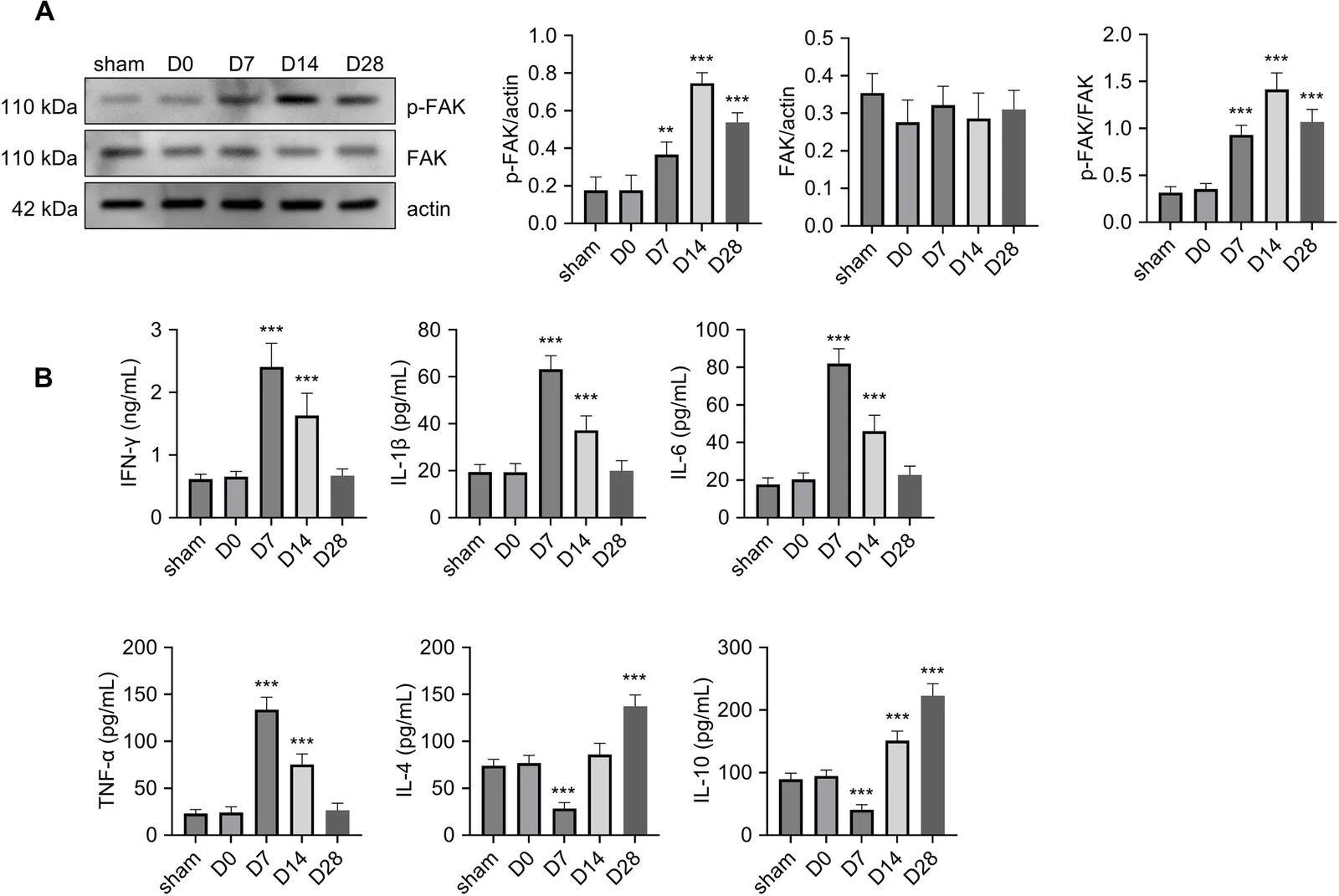
p-FAK is highly expressed during the proliferation-remodeling phase of fistula tract healing. (**A**) Western blot analysis of p-FAK and total FAK expression in fistula tract tissues at days 0, 7, 14, and 28 post-surgery. Bar graphs show quantification of p-FAK levels and p-FAK/FAK ratio. (**B**) Serum levels of pro-inflammatory cytokines (IFN-γ, TNF-α, IL-1β, IL-6) and anti-inflammatory cytokines (IL-4, IL-10) measured by ELISA at different time points. Data are presented as mean ± SD (*n* = 6 per group). *P < 0.05, **P < 0.01, ***P < 0.001 vs. day 0.

To define the temporal pattern of FAK activation during fistula tract maturation, quantitative Western blot analysis showed that total FAK protein levels remained relatively stable across all time points (days 0, 7, 14, and 28), whereas p-FAK levels began to rise significantly from day 7 post-surgery and remained elevated through days 14 and 28 (*P* < 0.01 compared to day 0) (Fig. 2A). The p-FAK/FAK ratio, reflecting FAK phosphorylation status, exhibited a similar temporal trend, with significant increases observed from day 7 onward (*P* < 0.001). This temporal pattern aligns with the classical phases of wound healing, as the peak of p-FAK expression (days 7–28) coincided with the transition from acute inflammation to the proliferative-remodeling phase. This suggests that FAK activation is not merely a transient stress response to initial injury but plays a sustained regulatory role in tissue repair and reconstruction processes. Measurement of inflammatory cytokines in serum samples further corroborated this healing progression. The early-stage post-injury period (days 0–7) was characterized by elevated pro-inflammatory factors (INF-γ, TNF-α, IL-1β, and IL-6) and relatively low anti-inflammatory factors (IL-4 and IL-10). During the mid-stage (days 7–14), pro-inflammatory cytokine levels gradually declined, while anti-inflammatory cytokines increased significantly, creating a more favorable microenvironment for tissue repair. By the late stage (days 14–28), pro-inflammatory cytokines were markedly reduced to near-baseline levels, and anti-inflammatory cytokines continued to rise, supporting tissue regeneration and resolution of inflammation (Fig. 2B).

### FAK activation potently drives proliferation and migration of intestinal mucosal epithelial cells

**Fig. 3:**
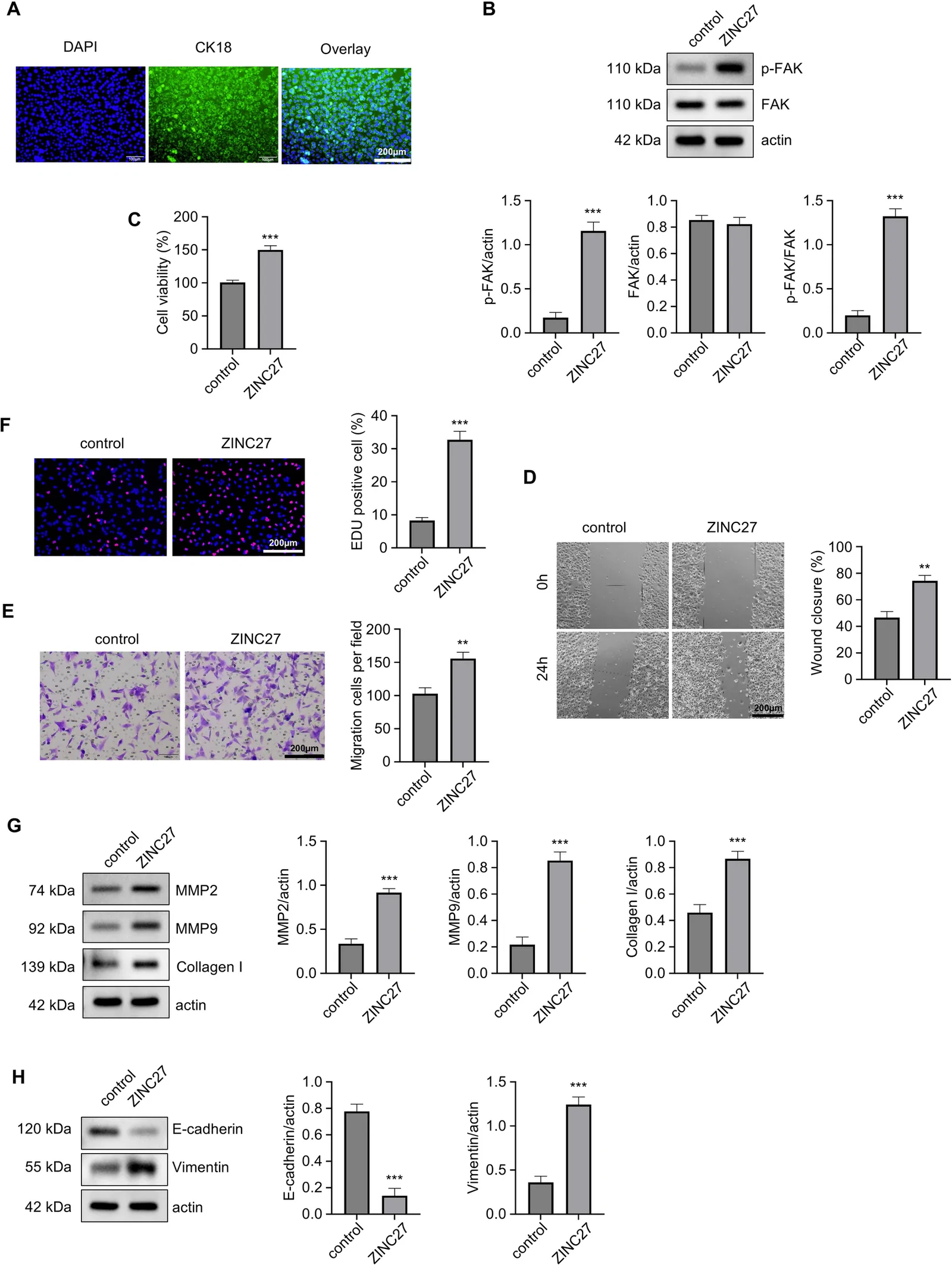
FAK activation potently drives proliferation and migration of intestinal mucosal epithelial cells. (**A**) Representative immunofluorescence images of primary intestinal epithelial cells stained with CK18 (green) and DAPI (blue). Scale bar = 200 μm. (**B**) Western blot analysis of p-FAK and total FAK expression in cells treated with ZINC27 (500 nM, 24 h) or vehicle control, with quantification of p-FAK/FAK ratio. (**C**) Cell viability assessed by CCK-8 assay in control and ZINC27-treated cells. (**D**) Representative images and quantification of EdU incorporation assay showing DNA synthesis in control and ZINC27-treated cells. EdU-positive cells (red), total nuclei (blue). Scale bar = 200 μm. (**E**) Representative images and quantification of scratch wound healing assay at 0 and 24 h. Scale bar = 200 μm. (**F**) Representative images and quantification of Transwell migration assay. Migrated cells were stained with crystal violet. Scale bar = 200 μm. (**G**) Western blot analysis of MMP2, MMP9, and Collagen I expression in control and ZINC27-treated cells, with quantification. (H) Western blot analysis of E-cadherin and Vimentin expression in control and ZINC27-treated cells, with quantification. Data are presented as mean ± SD from three independent experiments. **P < 0.01, ***P < 0.001 vs. control.

Primary intestinal mucosal epithelial cells were successfully isolated and their identity confirmed via cytokeratin 18 (CK18) immunofluorescence staining (Fig. 3A). Treatment with the FAK agonist ZINC40099027 (ZINC27, 500 nM for 24 h) selectively increased p-FAK levels without affecting total FAK expression, as confirmed by Western blot analysis (Fig. 3B). Functionally, ZINC27 treatment significantly enhanced cell viability as assessed by CCK-8 assay (Fig. 3C). Cell proliferation analyzed by the EdU incorporation assay demonstrated that ZINC27 significantly increased the percentage of EdU-positive cells compared to untreated controls, indicating enhanced DNA synthesis and cell proliferation (Fig. 3D). Cell migration assays demonstrated that ZINC27 treatment accelerated wound closure in scratch assays (Fig. 3E) and increased the number of cells migrating through Transwell inserts (Fig. 3F). Mechanistically, FAK activation upregulated MMP2, MMP9, and Collagen I protein levels (Fig. 3G) and induced an EMT-like phenotypic switch, evidenced by decreased E-cadherin and increased Vimentin expression (Fig. 3H). These results collectively indicate that FAK activation enhances both the proliferative and migratory capabilities of intestinal mucosal epithelial cells, promoting a pro-fibrotic phenotype that may contribute to fistula tract formation and persistence.

### FAK knockdown significantly inhibits proliferation and migration of intestinal mucosal epithelial cells

**Fig. 4:**
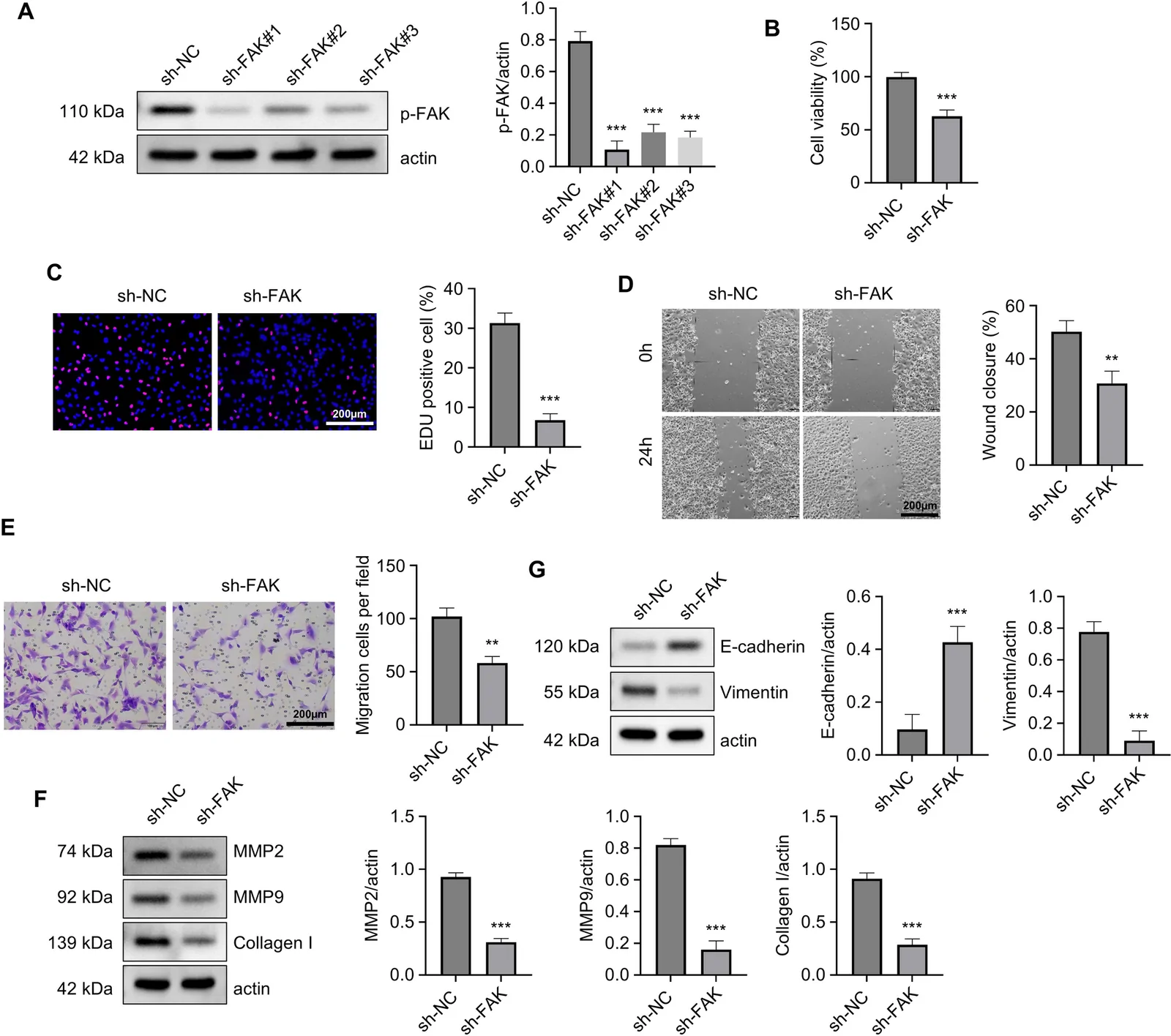
FAK knockdown significantly inhibits proliferation and migration of intestinal mucosal epithelial cells. (**A**) Western blot analysis confirming FAK knockdown efficiency using three different shRNA sequences (sh-FAK#1, #2, #3) compared to scrambled control (sh-NC), with quantification showing sh-FAK#1 achieved the highest knockdown efficiency. (**B**) Cell viability assessed by CCK-8 assay in sh-NC and sh-FAK#1 cells. (**C**) Representative images and quantification of EdU incorporation assay in sh-NC and sh-FAK#1 cells. EdU-positive cells (red), total nuclei (blue). Scale bar = 200 μm. (**D**) Representative images and quantification of scratch wound healing assay at 0 and 24 h in sh-NC and sh-FAK#1 cells. Scale bar = 200 μm. (**E**) Representative images and quantification of Transwell migration assay in sh-NC and sh-FAK#1 cells. Scale bar = 200 μm. (**F**) Western blot analysis of MMP2, MMP9, and Collagen I expression in sh-NC and sh-FAK#1 cells, with quantification. (**G**) Western blot analysis of E-cadherin and Vimentin expression in sh-NC and sh-FAK#1 cells, with quantification. Data are presented as mean ± SD from three independent experiments. **P < 0.01, ***P < 0.001 vs. sh-NC.

To further validate FAK function, lentiviral-mediated shRNA knockdown was employed. Western blot analysis confirmed effective knockdown of p-FAK by sh-FAK#1, #2 and #3, with sh-FAK#1 selected for subsequent experiments due to achieving the highest knockdown efficiency (Fig. 4A). FAK depletion significantly reduced cell viability (Fig. 4B) and markedly decreased the percentage of EdU-positive cells compared to sh-NC control (Fig. 4C). Additionally, FAK knockdown significantly inhibited cell migration, as demonstrated by both wound healing assays (Fig. 4D) and Transwell migration assays (Fig. 4E). At the molecular level, FAK knockdown decreased MMP2, MMP9, and Collagen I expression (Fig. 4F) and attenutaed the mesenchymal phenotype, with increased E-cadherin and decreased Vimentin levels (Fig. 4G). These loss-of-function findings were consistent with and complementary to the FAK activation experiments, demonstrating reciprocal effects and suggesting that FAK inhibition may represent a potential therapeutic strategy to prevent fistula tract formation.

### FAK acts as an upstream activator of the MAPK/ERK1 signaling pathway

**Fig. 5:**
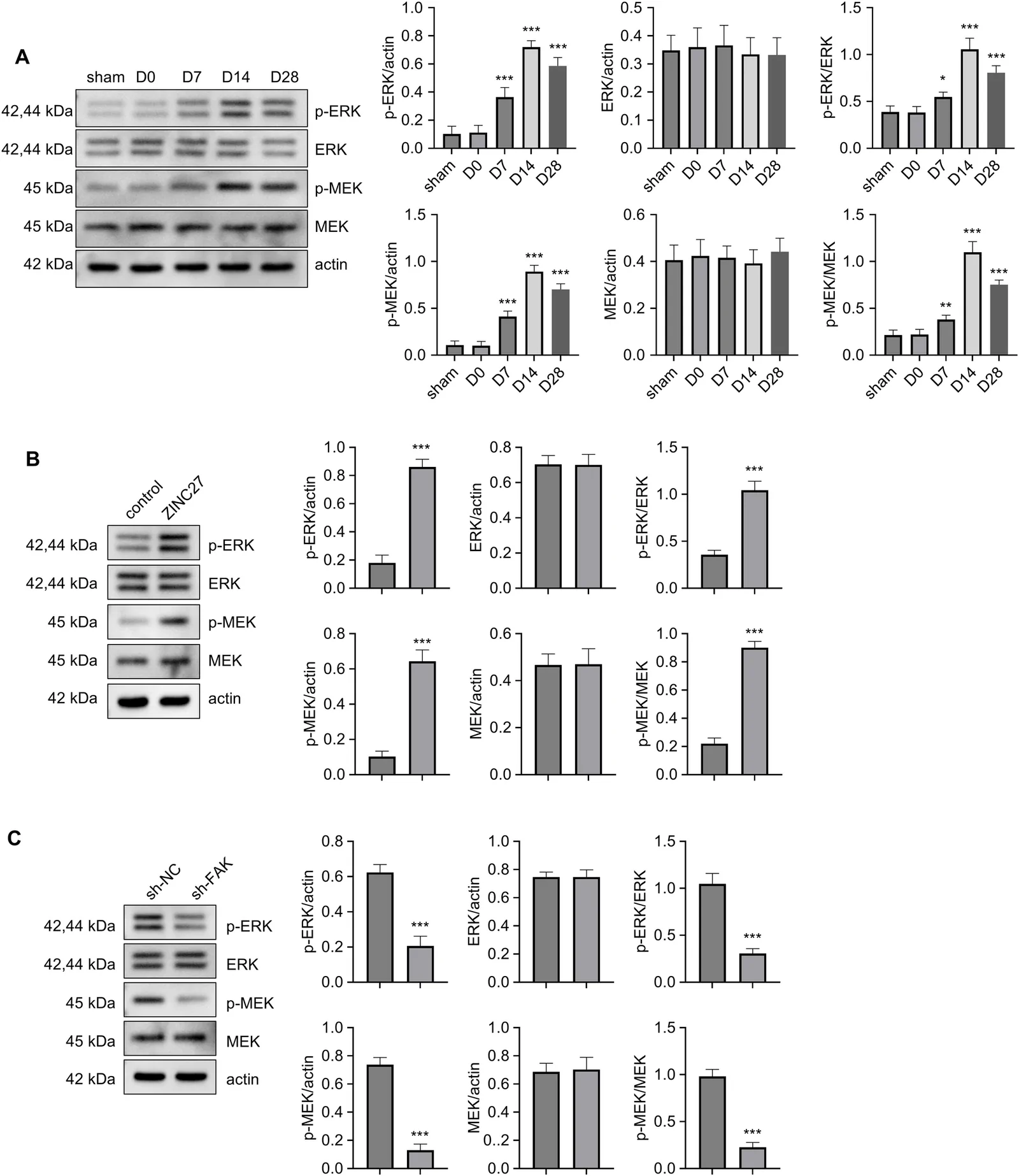
FAK acts as an upstream activator of the MAPK/ERK signaling pathway. (**A**) Western blot analysis of p-MEK1/2, total MEK1/2, p-ERK1/2, and total ERK1/2 expression in fistula tract tissues at days 0, 7, 14, and 28 post-surgery, with quantification of phosphorylation ratios (p-MEK/MEK and p-ERK/ERK). (**B**) Western blot analysis of p-MEK1/2, MEK1/2, p-ERK1/2, and ERK1/2 in control and ZINC27-treated cells, with quantification of phosphorylation ratios. (**C**) Western blot analysis of p-MEK1/2, MEK1/2, p-ERK1/2, and ERK1/2 in sh-NC and sh-FAK#1 cells, with quantification of phosphorylation ratios. Data are presented as mean ± SD (*n* = 6 per group for in vivo; *n* = 3 for in vitro). **P < 0.01, ***P < 0.001 vs. control or day 0.

To explore downstream signaling mechanisms mediating FAK-driven cellular responses, the MAPK/ERK signaling pathway was examined. In fistula tract tissues collected at different time points (days 0, 7, 14, and 28), phosphorylated p-MEK1/2 and p-ERK1/2 levels closely paralleled p-FAK expression, progressively increasing from day 7 and peaking at day 14, while total MEK1/2 and ERK1/2 levels remained relatively unchanged across all time points (Fig. 5A). This temporal concordance between FAK phosphorylation and MAPK/ERK activation suggests a potential regulatory relationship. In vitro, ZINC27 treatment of primary intestinal epithelial cells significantly upregulated p-MEK1/2 and p-ERK1/2 levels without affecting total protein expression, whereas FAK knockdown with sh-FAK#1 led to a significant reduction in p-MEK1/2 and p-ERK1/2 levels compared to sh-NC control (Fig. 5B, C). These results collectively indicate that FAK functions upstream of MAPK/ERK signaling, positively regulating its activation and suggesting that the FAK-MAPK/ERK axis plays a critical role in promoting intestinal epithelial cell proliferation, migration, and EMT-like phenotype during fistula tract formation.

### FAK mediates epithelial cell proliferation and migration via the MAPK/ERK signaling axis

**Fig. 6:**
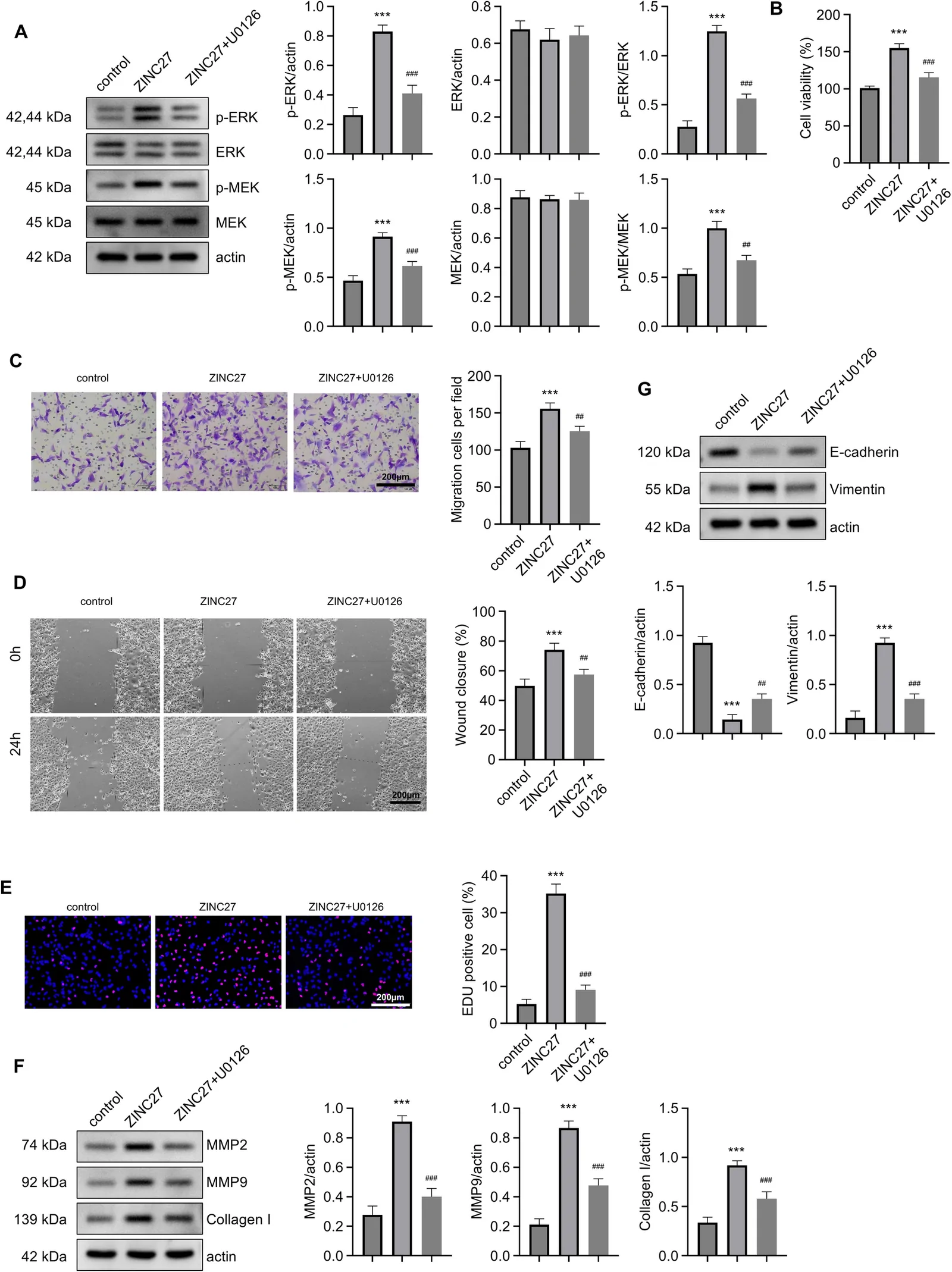
MEK/ERK inhibition abolishes FAK-mediated epithelial cell proliferation and migration. (**A**) Western blot analysis of p-MEK1/2, MEK1/2, p-ERK1/2, and ERK1/2 in cells treated with vehicle, ZINC27 (500 nM), or ZINC27 + U0126 (1 µM) for 24 h, with quantification. (**B**) Cell viability assessed by CCK-8 assay across treatment groups. (**C**) Representative images and quantification of EdU incorporation assay. Scale bar = 200 μm. (**D**) Representative images and quantification of scratch wound healing assay at 24 h. Scale bar = 200 μm. (**E**) Representative images and quantification of Transwell migration assay. Scale bar = 200 μm. (**F**) Western blot analysis of MMP2, MMP9, and Collagen I expression across treatment groups, with quantification. (**G**) Western blot analysis of E-cadherin and Vimentin expression across treatment groups, with quantification. Data are presented as mean ± SD from three independent experiments. Compared with the Control group, ***p < 0.001; compared with the ZINC27 group, ##*p* < 0.01; ###*p* < 0.001.

**Fig. 7:**
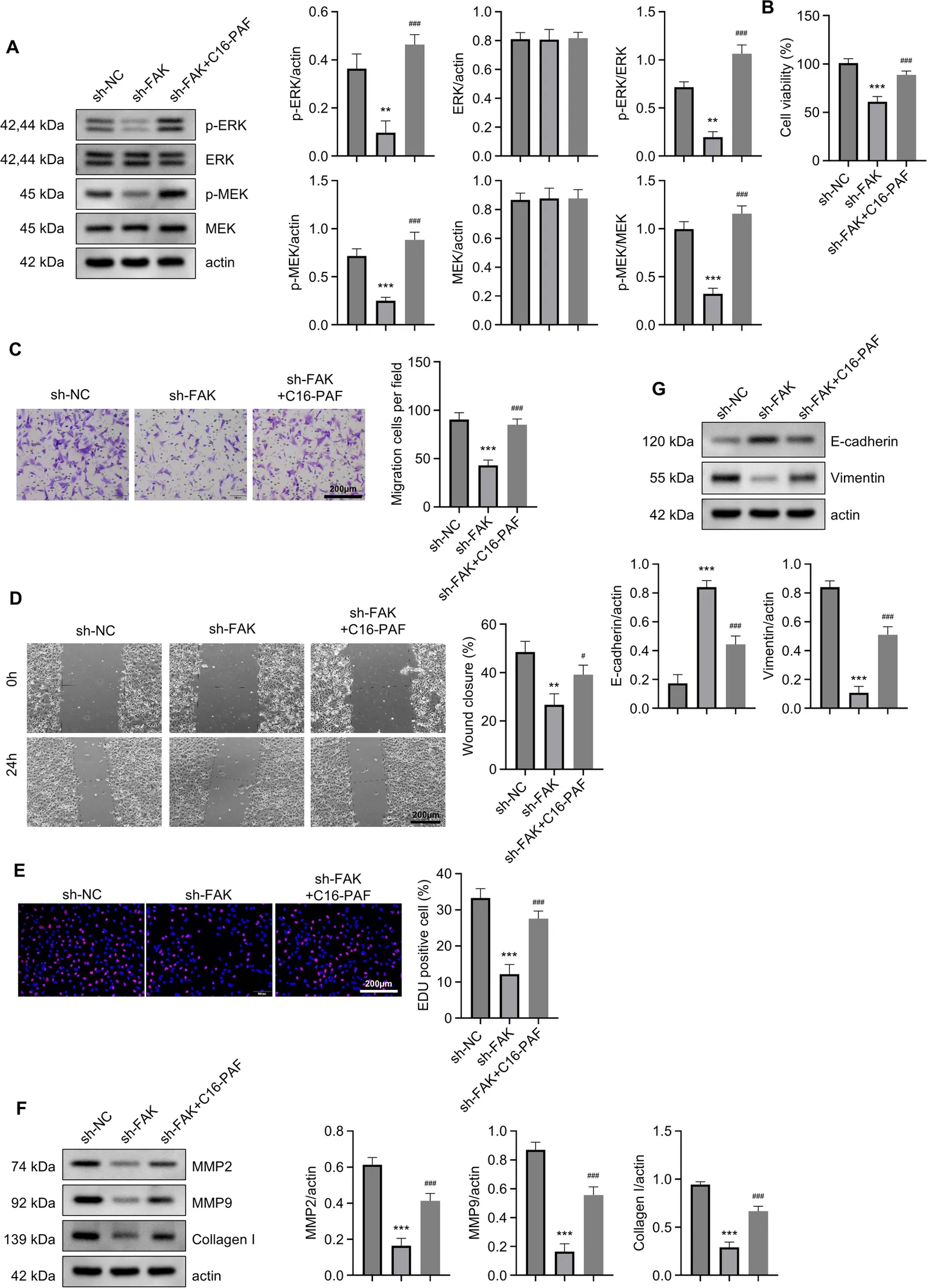
MEK/ERK activation rescues impaired proliferation and migration in FAK-knockdown cells. (**A**) Western blot analysis of p-MEK1/2, MEK1/2, p-ERK1/2, and ERK1/2 in sh-NC cells, sh-FAK#1 cells, and sh-FAK#1 cells treated with C16-PAF (5 µM, 24 h), with quantification. (**B**) Cell viability assessed by CCK-8 assay across groups. (**C**) Representative images and quantification of EdU incorporation assay. Scale bar = 200 μm. (**D**) Representative images and quantification of scratch wound healing assay at 24 h. Scale bar = 200 μm. (**E**) Representative images and quantification of Transwell migration assay. Scale bar = 200 μm. (**F**) Western blot analysis of MMP2, MMP9, and Collagen I expression across groups, with quantification. (**G**) Western blot analysis of E-cadherin and Vimentin expression across groups, with quantification. Data are presented as mean ± SD from three independent experiments. Compared with the sh-NC group, **p < 0.01; ***p < 0.001; compared with the sh-FAK group, #*p* < 0.05; ##*p* < 0.01; ###*p* < 0.001.

Bidirectional rescue experiments were conducted to establish causality between FAK and MAPK/ERK signaling in regulating cellular functions. In ZINC27-treated cells, co-administration of the MEK1/2 inhibitor U0126 (1 µM for 24 h) effectively blocked ZINC27-induced p-MEK1/2 and p-ERK1/2 elevation (Fig. 6A), and abrogated the pro-proliferative effects as measured by CCK-8 assay (Fig. 6B) and EdU incorporation assay (Fig. 6C), and the pro-migratory effects of ZINC27 in both wound healing and Transwell assays (Fig. 6D, E). Upregulation of MMP2, MMP9, and Collagen I, and EMT marker changes (decreased E-cadherin and increased Vimentin) induced by ZINC27 were also reversed by U0126 (Fig. 6F, G). Conversely, in FAK-knockdown cells, the MEK/ERK activator agonist C16-PAF (5 µM for 24 h) restored p-MEK1/2 and p-ERK1/2 levels (Fig. 7A), and rescued the impaired proliferation as assessed by CCK-8 assay (Fig. 7B) and EdU incorporation assay (Fig. 7C), and enhanced migration in wound healing and Transwell assays (Fig. 7D, E). MEK/ERK activation also partially restored downstream protein expression (MMP2, MMP9, Collagen I, E-cadherin, and Vimentin) (Fig. 7F, G). These results collectively confirm that the MAPK/ERK signaling pathway mediates FAK-dependent regulation of intestinal epithelial cell proliferation, migration, and EMT-like phenotypic changes.

### In vivo FAK knockdown delays healing and maturation of rabbit drainage tube-induced fistula tracts

**Fig. 8:**
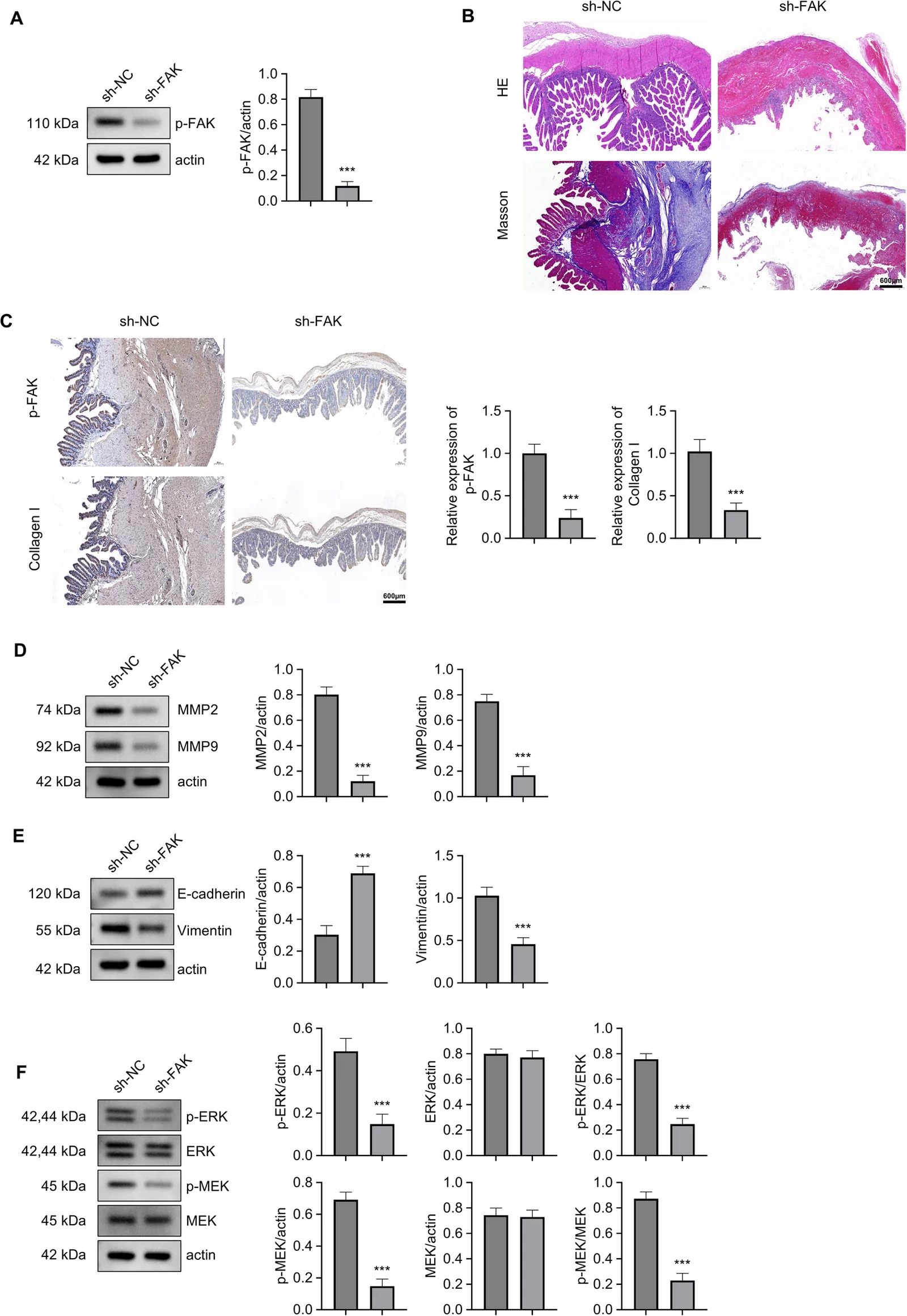
In vivo FAK knockdown delays healing and maturation of rabbit drainage tube-induced fistula tracts. (**A**) Western blot analysis confirming FAK knockdown in fistula tract tissues from rabbits treated with sh-NC or sh-FAK adenovirus, with quantification. (**B**) Representative images of H&E staining and Masson’s trichrome staining of fistula tract tissues from sh-NC and sh-FAK groups at day 14 post-surgery. Scale bars = 600 μm. (**C**) Representative immunohistochemical images showing p-FAK and Collagen I expression in fistula tract tissues from sh-NC and sh-FAK groups. Scale bars = 600 μm. (**D**) Western blot analysis of MMP2 and MMP9 expression in fistula tract tissues from sh-NC and sh-FAK groups, with quantification. (**E**) Western blot analysis of E-cadherin and Vimentin expression in fistula tract tissues from sh-NC and sh-FAK groups, with quantification. (**F**) Western blot analysis of p-MEK1/2, MEK1/2, p-ERK1/2, and ERK1/2 expression in fistula tract tissues from sh-NC and sh-FAK groups, with quantification of phosphorylation ratios. Data are presented as mean ± SD (*n* = 6 per group). **P < 0.01, ***P < 0.001 vs. sh-NC.

To validate these findings at the whole-animal level, sh-FAK adenovirus was locally injected into the intestinal wall and surrounding mesenteric tissues during model establishment, achieving specific FAK knockdown in fistula tract tissue as confirmed by Western blot analysis (Fig. 8A). At two weeks post-surgery, histological analysis by H&E staining revealed that the sh-FAK group displayed thinner and more loosely organized fistula tract walls, with markedly reduced granulation tissue formation compared to the sh-NC group (Fig. 8B). Masson’s trichrome staining demonstrated markedly reduced collagen fiber deposition and disorganized collagen architecture in sh-FAK-treated animals compared to the sh-NC group (Fig. 8B). IHC analysis of fistula tract tissues confirmed decreased levels of p-FAK and Collagen I in sh-FAK-treated tissues (Fig. 8C). Moreover, Western blot analyses showed reduced levels of MMP2, MMP9, and Vimentin, as well as reduced phosphorylation of MEK1/2 and ERK1/2 after FAK knockdown, whereas E-cadherin expression was significantly elevated (Fig. 8D–F). These in vivo results were consistent with in vitro findings, demonstrating that FAK silencing disrupts the coordinated progression of tissue remodeling and results in delayed healing and incomplete fistula tract formation, suggesting an essential role of FAK-MAPK/ERK signaling axis in fistula tract healing and repair.

## Discussion

The formation and healing of postoperative drainage tube-induced abdominal fistula tracts represent highly dynamic tissue repair processes that directly impact patient outcomes^23,24^. In this study, we systematically investigated the molecular mechanisms underlying fistula tract maturation and identified FAK as a pivotal regulator promoting intestinal epithelial cell proliferation, migration, and extracellular matrix remodeling through MAPK/ERK signaling activation. Using a rabbit fistula model combined with in vitro primary epithelial cell cultures and bidirectional rescue experiments, we demonstrated that FAK phosphorylation increases progressively during the transition from inflammation to proliferation-remodeling phases. Mechanistically, FAK functions upstream of MEK/ERK signaling to drive epithelial-mesenchymal transition, matrix metalloproteinase expression, and collagen deposition—key events essential for fistula tract stabilization. Importantly, local FAK knockdown in vivo significantly impaired fistula tract maturation, resulting in reduced collagen deposition, disorganized tissue architecture, and persistent inflammation. These findings advance our understanding of abnormal tissue repair in fistula formation and identify the FAK-MAPK/ERK axis as a potential therapeutic target for managing refractory enterocutaneous fistulas.

A cornerstone of this study was the successful establishment of a rabbit drainage tube-induced intestinal fistula tract model that faithfully recapitulated the pathological progression from acute inflammation through granulation tissue formation, fibrotic remodeling, and eventual epithelialization. Critically, activated FAK (p-FAK) expression did not peak during the initial inflammatory phase (days 0–7) but increased significantly from day 7 to day 28, coinciding with inflammation resolution and the transition to proliferative-remodeling phases. This temporally restricted pattern indicates that FAK is not merely an immediate stress-response molecule but functions as a central regulator during the tissue repair execution phase. Nascent extracellular matrix components, particularly Collagen I, likely activate FAK through integrin engagement, while growth factors released at the wound site may synergistically amplify this response^25–27^. In this context, FAK functions as an integrator of intracellular and extracellular signals^28^, with its timely activation serving as a molecular switch orchestrating subsequent cellular behaviors essential for organized tissue repair and fistula tract maturation.

A key finding of this study is the confirmation of FAK’s critical regulatory role in intestinal epithelial cell proliferation and migration. Both gain-of-function (ZINC27 agonist) and loss-of-function (shRNA knockdown) experiments demonstrated that FAK is both necessary and sufficient to drive these cellular processes. Notably, FAK activation induced EMT-like molecular changes, characterized by E-cadherin downregulation and Vimentin upregulation, markers commonly observed during epithelial plasticity and phenotypic transitions. EMT is widely recognized as a critical mechanism enabling epithelial cells to acquire migratory and invasive capabilities, playing central roles in embryonic development, organ fibrosis, and cancer metastasis^29–32^. In the context of intestinal epithelial biology, EMT-like molecular signatures have been extensively documented in inflammatory bowel disease and Crohn’s disease, where epithelial cells exhibit partial loss of epithelial markers and acquisition of mesenchymal characteristics^33,34^. Buckley and colleagues demonstrated that intestinal epithelial cells are capable of undergoing EMT-associated molecular changes in vitro, displaying altered expression of epithelial and mesenchymal markers under specific stimuli^35^. Our results directly link FAK activation to these EMT-like molecular changes in rabbit intestinal epithelial cells, suggesting that FAK serves as an upstream regulator initiating this phenotypic reprogramming. The observed E-cadherin downregulation would be expected to reduce epithelial cell-cell adhesion strength, while Vimentin upregulation indicates acquisition of mesenchymal-like cytoskeletal organization, both contributing to enhanced cellular motility. Furthermore, FAK-induced upregulation of MMP2 and MMP9 facilitates ECM remodeling, creating permissive conditions for cell migration^36,37^, consistent with our functional observations of enhanced epithelial cell mobility and fistula tract maturation. Whether these EMT-like changes represent transient, reversible phenotypic plasticity or sustained pathological transitions remains to be fully elucidated and warrants future investigation using temporal analysis of mesenchymal-to-epithelial transition (MET) markers.

A significant contribution of this study lies in establishing the MAPK/ERK signaling pathway as a critical downstream mediator of FAK function. We observed that p-MEK1/2 and p-ERK1/2 levels consistently paralleled p-FAK expression in both in vivo fistula tract tissues and in vitro primary epithelial cell systems. The MAPK/ERK pathway is a classic regulator of cell proliferation and migration and is indispensable in these processes^38,39^. To establish a causal relationship between FAK and MAPK/ERK activation, we performed bidirectional rescue experiments. Pharmacologic inhibition using the MEK inhibitor in ZINC27-treated cells demonstrated that MAPK/ERK activation is necessary for FAK function, as pathway blocking abolished FAK-mediated proliferation, migration, and EMT-like changes. Conversely, MEK/ERK agonist rescue (C16-PAF treatment in sh-FAK cells) demonstrated sufficiency, as direct activation of MAPK/ERK signaling restored cellular function despite upstream FAK deficiency. These complementary experiments definitively establish the MAPK/ERK pathway as a key effector through which FAK exerts its biological effects on intestinal epithelial cell behavior during fistula tract formation.

Critically, adenovirus-mediated FAK knockdown in the rabbit fistula tract model recapitulated our in vitro findings, demonstrating delayed healing, suppressed MAPK/ERK signaling, and altered tissue architecture, thereby strengthening the translational relevance of our conclusions. Impaired epithelialization and aberrant tissue remodeling represent central pathological features of refractory fistula tracts, including complex perianal fistulas in Crohn’s disease and enterocutaneous fistulas following radiation enteritis^40,41^. Given FAK’s role in coordinating epithelial migration and extracellular matrix remodeling, therapeutic strategies employing FAK modulators may offer innovative approaches for promoting fistula closure. However, several mechanistic questions remain. The upstream triggers of FAK activation—whether mechanical tension, growth factor signaling, integrin-ECM interactions, or their combinations—require further clarification. Additionally, while we identified MAPK/ERK as a key effector, FAK likely engages other pathways, such as PI3K/AKT and Rho GTPases, which may contribute through parallel or convergent mechanisms. Future studies employing comprehensive phosphoproteomic analyses will be essential to map the complete FAK signaling network and identify optimal therapeutic intervention points for clinical translation.

## Conclusion

In conclusion, this study employed an integrated in vivo and in vitro experimental approach to systematically demonstrate that FAK drives intestinal epithelial cell proliferation, migration, and EMT-like phenotypic changes through MAPK/ERK signaling activation, thereby promoting fistula tract maturation and healing. Using a clinically relevant rabbit drainage tube-induced fistula model combined with primary epithelial cell cultures and bidirectional rescue experiments, we established FAK as an upstream regulator and MAPK/ERK as a critical downstream effector in coordinating epithelialization and extracellular matrix remodeling. These findings advance our mechanistic understanding of abnormal tissue repair processes and identify the FAK-MAPK/ERK axis as a promising therapeutic target for managing refractory enterocutaneous fistulas in clinical practice.

## Supplementary Information

Below is the link to the electronic supplementary material.

Supplementary Material 1

## Data Availability

The datasets generated during and/or analysed during the current study are available from the corresponding author on reasonable request.
